# Mesoscale maladaptation in disease organoids

**DOI:** 10.1242/dmm.052951

**Published:** 2026-06-29

**Authors:** Masashi Okamoto, Takanori Takebe

**Affiliations:** ^1^ Department of Genome Biology, Graduate School of Medicine, and Premium Research Institute for Human Metaverse Medicine (WPI-PRIMe), the University of Osaka, Suita, Osaka 565-0871, Japan; ^2^ Department of Respiratory Medicine and Clinical Immunology, Graduate School of Medicine, the University of Osaka, Suita, Osaka 565-0871, Japan; ^3^ Human Biology Research Unit, Institute of Integrated Research, Institute of Science Tokyo (Science Tokyo), 1-5-45, Yushima, Bunkyo-ku, Tokyo 113-8510, Japan; ^4^ Division of Gastroenterology, Hepatology and Nutrition & Division of Developmental Biology, Cincinnati Children's Hospital Medical Center, 3333 Burnet Avenue, Cincinnati, OH 45229-3039, USA; ^5^ The Center for Stem Cell and Organoid Medicine (CuSTOM), Cincinnati Children's Hospital Medical Center, 3333 Burnet Avenue, Cincinnati, OH 45229-3039, USA; ^6^ Department of Pediatrics, University of Cincinnati College of Medicine, 3333 Burnet Avenue, Cincinnati, OH 45229-3039, USA; ^7^ Communication Design Center, Advanced Medical Research Center, Yokohama City University, Yokohama, Kanagawa 236-0004, Japan

## Abstract

In chronic diseases, multiple tissue components at a shared interface often deteriorate concurrently, and disease progression may depend on interactions among these failures rather than on any single defect. Multi-tissue organoid models can mimic disease-relevant pathology *in vitro*, but the field lacks a simple framework for comprehensive interrogation. Here, we propose mesoscale maladaptation as an operational concept for multi-tissue disease modelling, defined as a synergistic decline in interdependent functions that can occur between two or more tissue elements. To detect maladaptation, we introduce a stepwise workflow that identifies the failing tissue elements, defines directionality of potential interdependence between these elements and compares combined perturbations with single perturbations to evaluate synergistic decline. We apply this framework to intestinal neuromuscular, hepatic sinusoidal, blood–brain barrier and tumour–neural interfaces, but it can be extrapolated to other organs and systems. This framework will illuminate future directions for *in vitro* complex disease modelling by enriching biological insights to disentangle progressive pathology, shifting the focus from localised biological failures to concurrent multi-tissue failures that produce synergistic pathology.

## Teaser

**Summary:** This Perspective proposes mesoscale maladaptation as a framework for interrogating concurrent failures across multi-tissue organoid interfaces that create disease-relevant, synergistic pathology across lineages.

## Introduction

When pathology is confined to a single cell lineage, single-lineage models can identify the causal defect. In many chronic inflammatory, metabolic and fibrotic diseases, however, failure is often not restricted to one cell type. Instead, multiple tissue components at a shared interface deteriorate in parallel, and pathology arises from their interactions rather than from any single defect. For example, in metabolic dysfunction-associated steatotic liver disease (MASLD), perturbations, such as hepatocyte lipotoxicity, sinusoidal capillarisation and stellate cell activation, can develop in parallel, influenced by converging genetic susceptibility, epigenetic reprogramming, and exposure factors such as dietary metabolites and xenobiotics (Kimura et al., 2022; Sillé et al., 2025). A hepatocyte-only culture can model lipotoxicity, but it cannot test whether changes in the neighbouring sinusoid exacerbate lipotoxic stress into a distinct phenotype. Capturing this kind of interaction-driven pathology, which arises from concurrent multi-tissue dysfunction, is a central strength of multi-tissue organoid models. This is especially important in chronic disease, as such diseases rarely arise from a single acute injury. Instead, such pathology builds up over years, as low-grade injury accumulates, neighbouring tissues keep compensating, and the interface itself slowly remodels. If multi-tissue organoid models can help us identify which node fails first, how that failure spreads to its neighbours and which combined perturbations synergistically worsen interface function, they can become useful tools to understand, predict, and intervene in chronic interface pathology.

Recent advances in stem cell and organoid technologies have enabled the self-organisation of multi-tissue constructs equipped with tissue–tissue interfaces *in vitro* (Clevers, 2016; Takebe and Wells, 2019). Multi-tissue constructs are increasingly reported to exhibit inter-tissue-dependent functional outputs (Workman et al., 2017; Saiki et al., 2025; Kang et al., 2025; Dao et al., 2024). These systems hold enormous potential for modelling complex disease because they contain multi-lineage tissues in which concurrent, progressive failures may interact and shape pathology. However, the field still lacks a shared framework for assessing whether such interactions occur and, if they do, whether their combined effects are simply additive or deviate from an expected combined effect.

**Fig. 1.**
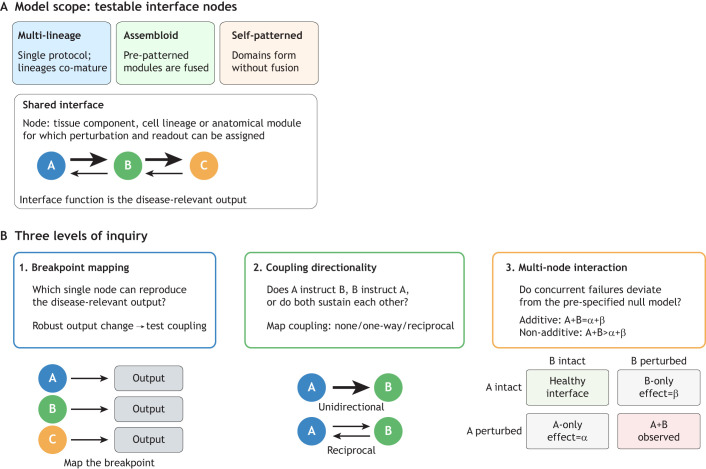
**Mesoscale maladaptation.** (A) Multi-tissue organoids include multi-lineage organoids (a single protocol allows several lineages to co-differentiate), multi-organ assembloids (pre-patterned modules are intentionally fused) and self-patterned multi-organ organoids (multiple regions arise without explicit fusion). In each format, the construct contains interacting tissue nodes for which combined state at a shared interface determines a disease-relevant interface output. (B) Chain dissection progresses through three levels of inquiry: (1) breakpoint mapping asks which single-node perturbation reproduces the disease-relevant output; (2) coupling directionality asks whether dependency between nodes is unidirectional or reciprocal; and (3) multi-node interaction compares single and combined perturbations against a pre-specified null model and is the level that directly tests for mesoscale maladaptation. The 2×2 perturbation matrix in B(3) compares the observed combined effect in the lower-right cell against a pre-specified additive or multiplicative null derived from the single-node effects α and β.

Multi-tissue organoid models can be generated in several ways (Fig. 1A). In multi-lineage organoids, a differentiation protocol allows multiple germ layers or lineages to co-mature within one construct. In assembloids, pre-patterned modules are intentionally fused to create a defined interface. In self-patterned organoids, multiple tissue- or organ-like domains emerge even without explicit fusion. These formats differ in experimental control, maturation and interface geometry, but all create opportunities to ask whether disease-relevant outputs depend on interactions among neighbouring tissues rather than on one compartment alone.

‘Mesoscaling’ is a potentially valuable framework to understand such complex tissue organisation and remodelling processes. Shao and Fu (2022) defined this vocabulary to describe how multiple microscale units structurally organise and interact within and across tissues. Chen et al. (2025) proposed a complementary concept of mesoscale modules to understand recurring multicellular functional units with associated parts lists and constraints. Building upon these structural foundations, we here propose mesoscale maladaptation as an operational concept oriented toward tissue malfunction and disease modelling. The concept is, in a way, analogous to epistasis and synthetic lethality because all ask whether combined perturbations produce outcomes that cannot be predicted from single perturbations alone (Phillips, 2008; Kaelin, 2005). However, mesoscale maladaptation is framed at the level of interacting tissue nodes in an *in vitro* synthetic tissue model, uses interface function as the output, and focuses on disease-relevant decline rather than genetic fitness or cancer-cell viability. Compared with the broader phrase ‘emergent multi-tissue pathology’, our term adds an operational test with defined nodes, a measured interface output and a pre-specified null model for combined perturbations. We then describe a strategy for detecting this maladaptation, which we term chain dissection, and apply it to three exemplar tissue interfaces.

We refer to this condition as mesoscale maladaptation: a decline in interface function that arises when two or more tissue nodes within a multi-lineage boundary are simultaneously dysfunctional and produce a combined output that cannot be explained by studying any node alone

## Defining ‘mesoscale maladaptation’

In this Perspective, we use the term ‘node’ to mean a disease-relevant tissue component or cell lineage within an interface. In many chronic diseases, pathology does not arise from a single cellular defect, but from the concurrent dysfunction of multiple nodes at a shared boundary. For example, in inflammatory bowel disease, epithelial barrier disruption, endothelial activation and immune-cell recruitment can occur together in response to microbial and environmental triggers (Sillé et al., 2025), and blocking the α4β7 integrin–mucosal addressin cell adhesion molecule 1 (MAdCAM-1) homing axis can alter this interface clinically (Sandborn et al., 2013). Disease progression may, therefore, reflect interactions among concurrent failures rather than the effect of any single dysfunctional node, and targeting these interactions therapeutically presents an attractive clinical opportunity. We refer to this condition as mesoscale maladaptation: a decline in interface function that arises when two or more tissue nodes within a multi-lineage boundary are simultaneously dysfunctional and produce a combined output that cannot be explained by studying any node alone. In this Perspective, non-additive decline refers to a combined perturbation outcome that exceeds the response predicted from the individual perturbations under a pre-specified null model. To make this concrete, we consider an interface with two (or more) candidate tissue nodes and label them A and B. The simplest null is additive: under this null, the expected combined effect equals the effect of perturbing node A alone plus the effect of perturbing node B alone. We mainly use mesoscale maladaptation for cases in which the combined effect of A and B is greater than the sum of A alone plus B alone. Therefore, combined perturbation causes disproportionate worsening, or a pre-specified categorical state shift, at the interface. Antagonistic non-additivity can still reveal coupling between nodes, but we regard such cases as failed compensation (mesoscale maladaptation) only when they worsen interface function or expose a disease-relevant failure mode (Fig. 1).

Mesoscale maladaptation can be organised into three progressively deeper levels of inquiry (Fig. 1B):

1. Breakpoint mapping. When a functional output is reduced or lost, which single node represents the primary site of failure? This question can be addressed by applying node-selective perturbations and observing which intervention reproduces the disease phenotype.
2. Coupling directionality. Is the dependency between two nodes unidirectional (A instructs B) or reciprocal (A and B sustain each other)? In cases of reciprocal coupling, dysfunction at either node can destabilise the other, so the interface itself becomes the disease-relevant unit rather than any single cell type.
3. Multi-node interaction. When two or more nodes are perturbed simultaneously, does the resulting phenotype deviate disproportionately, or as a pre-specified categorical state shift, from the sum of individual disruptions? Addressing this question requires combinatorial perturbation designs in which nodes are disrupted independently, like they would be for testing breakpoint mapping and coupling directionality, but also in combination. If the combined phenotype deviates from the pre-specified null model, this constitutes evidence of non-additive interaction, and thus mesoscale maladaptation.

These three levels are connected but not equivalent. Breakpoint mapping identifies candidate failing nodes for a defined primary interface output. Coupling directionality then tests whether failure propagates from one node to another or in both directions. These first two levels make the final test interpretable, but they do not by themselves establish mesoscale maladaptation. Multi-node interaction is the decisive test, because it compares single and combined perturbations against a pre-specified null model and asks whether concurrent failures produce a disease-relevant output that deviates from the expected combined effect.

To address these queries systematically, we discuss a chain dissection approach in the following section. This describes the use of selective perturbations to identify which node, or combination of nodes, within a multi-tissue interface drives a disease-relevant change.

## Mesoscale maladaptation: an analytical framework

**Fig. 2.**
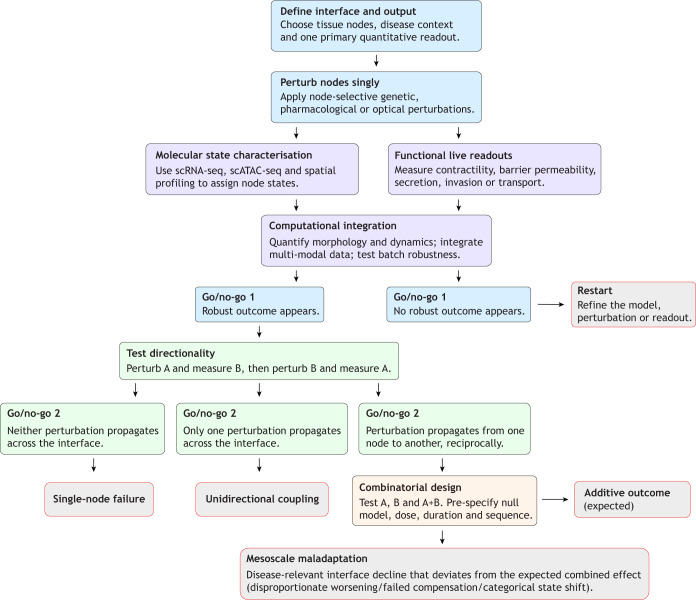
**Decision workflow for applying chain dissection: define interface, candidate nodes and a quantitative output; perturb each node singly to map breakpoints using mechanistic state characterisation and/or functional live readouts, with computational techniques for analysis.** Molecular state characterisation, functional live readouts and computational analysis can be repeated and/or tailored for downstream testing of directionality and combinatorial design. Go/no-go 1: refine the model if no robust effect appears; or if a robust outcome appears, move on to testing directionality. Go/no-go 2: during directionality testing, if neither perturbation propagates across the interface it can be classified as a single-node failure, if only one perturbation propagates across the interface it can be classified as unidirectional coupling but if perturbation propagates from one node to another, reciprocally, then move on to combinatorial design. Combined-output classification: additive (expected) or one of three maladaptive patterns (disproportionate worsening/failed compensation/categorical state shift). scATAC-seq, single-cell assay for transposase-accessible chromatin sequencing; scRNA-seq, single-cell RNA sequencing.

Detecting maladaptation through chain dissection relies on three complementary classes of technology (Fig. 2). The first is node-selective perturbation. Lineage-restricted genetic engineering, for example introducing a disease variant into one induced pluripotent stem cell (iPSC)-derived compartment while maintaining the other as wild type, allows cell-autonomous and non-cell-autonomous effects to be distinguished (Geurts and Clevers, 2023). Pharmacological agents, blocking antibodies and optogenetic tools also provide temporally controllable alternatives for targeting individual nodes. These techniques can be harnessed individually and in combination to interrogate mesoscale maladaptation at its three levels of breakpoint mapping, coupling directionality and multi-node interaction. The second class of technology enables molecular state characterisation following the use of these node-selective perturbations. Single-cell RNA sequencing (scRNA-seq) and single-cell assay for transposase-accessible chromatin sequencing (scATAC-seq) can define the transcriptional and chromatin state of each node before and after perturbation, distinguishing direct effects from secondary responses propagated across the interface. Furthermore, spatial transcriptomics adds positional resolution, mapping gene expression changes at the boundary between interacting tissues. Because each measurement captures a snapshot at a single timepoint, it reveals the state of each node but not the dynamics of how perturbation signals propagate across the interface. Comparing snapshots taken at multiple intervals after a node-selective perturbation can partially reconstruct this sequence, and trajectory inference methods, such as RNA velocity, provide additional resolution by inferring directional state transitions from a single timepoint. Nonetheless, these molecular readouts are most powerful when paired with the third class of technology that enables functional live readouts, which capture dynamic coupling in real time. Genetically encoded calcium indicators (e.g. GCaMP), voltage sensors and video-based contraction analysis enable real-time monitoring of neural, electrical, and mechanical activity in neuromuscular chains. Fluorescent barrier probes (e.g. FITC-dextran) quantify epithelial and endothelial integrity, while metabolic assays, such as cholyl-lysyl-fluorescein clearance and albumin secretion, measure hepatocyte transport and synthetic function. Functional readouts are rarely node specific by themselves: fluorescent probes report barrier integrity only when the signal is spatially assigned to the relevant epithelial or endothelial interface, and metabolic assays are node informative when the measured function is lineage restricted, such as hepatocyte transport or secretion. These readouts become node informative when paired with spatial localisation, lineage reporters or cell-type-specific sensors, and they become indicators of inter-node coupling when measured before and after perturbation of a neighbouring node.

**Figure:**
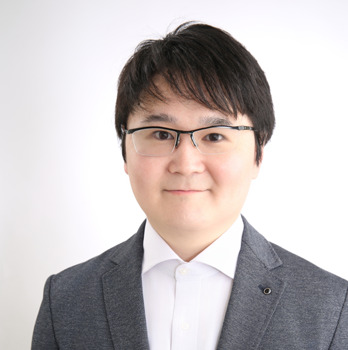
Masashi Okamoto

Unlike standard experimental practice, chain dissection requires that the same interface be examined through a defined sequence of perturbations. First, the investigator defines the interface, candidate nodes and a quantitative interface output before perturbation. Second, each node is perturbed individually and in combination to examine the three levels of mesoscale maladaptation: breakpoint mapping, coupling directionality and multi-node interaction. This sequence provides practical go/no-go criteria: if individual perturbations do not affect the output, the readout or node definition should be revised; if neither perturbation propagates across the interface, affecting a neighbouring node, the system may represent single-node failure; if only one direction propagates, the interface should be analysed as unidirectional coupling; if combined perturbation produces a disproportionate or new pathological state, the interface becomes the disease-relevant unit of analysis (Fig. 2).

Computational analysis should be matched to the level of inquiry. For breakpoint mapping, image segmentation and high-content phenotyping can quantify which single-node perturbation best reproduces the disease-relevant output. For coupling directionality, multimodal integration of spatial transcriptomics, single-cell state changes and live functional readouts can help identify whether perturbation effects propagate from node A to node B, from node B to node A, or reciprocally. For multi-node interaction, the key computational task is interaction scoring: quantifying the deviation of the combined-perturbation outcome from the pre-specified null model. Machine-learning tools can then help integrate morphology, molecular state and functional dynamics into a composite output, reported either as a continuous interaction score or as a categorical phenotype class. Because these designs generate high-dimensional morphological, molecular and functional datasets, artificial intelligence (AI)-assisted phenotypic analysis will become increasingly important as this organ-agnostic framework is applied to the expanding repertoire of multi-tissue organoid and assembloid systems. Predictive tools for combinatorial perturbations may be useful, but they should be treated as hypothesis generating unless validated by direct perturbation experiments (Bai et al., 2024; Maramraju et al., 2024; Roohani et al., 2024). For translational use, these models should report calibration, batch sensitivity and validation across independent organoid lines before their predictions are treated as evidence of interaction. Computational tools should prioritise perturbation combinations and quantify interaction scores, but causal claims should remain anchored in direct perturbation and rescue experiments.

## Three exemplars: from single-node failure to mesoscale maladaptation

We apply this framework to three exemplar interfaces. The first demonstrates breakpoint mapping in the context of single-node failure at the intestinal-neuromuscular interface. The second highlights interface-dependent coupling consistent with reciprocal signalling in the liver sinusoid and proposes a platform for testing mesoscale maladaptation. The third applies the framework to neural organoid and assembloid systems, where blood–brain barrier (BBB) and tumour–neural interfaces provide concrete examples of multi-node interaction. Table 1 extends the framework to additional organ–system interfaces, suggesting that the logic applies beyond the three exemplars discussed here.

**Table 1.** Applying chain dissection across organ–system interfaces

Interface	Tissue nodes	Disease context	Perturbation logic and readouts	Key references
Intestine–neuromuscular	Epithelium, ENS, ICC, smooth muscle	Hirschsprung disease; dysmotility	TTX, Methylene Blue and PHOX2B perturbations map distinct breakpoints; contraction is the output.	Workman et al., 2017; Childs et al., 2025
Liver–sinusoidal	Hepatocyte, LSEC, stellate cell	MASLD, capillarisation and fibrosis	WNT2 perturbation tests reciprocal coupling; lipotoxicity plus capillarisation can test non-additive fibrotic output.	DeLeve et al., 2004; Gracia-Sancho et al., 2021; Saiki et al., 2025
Intestine–vascular–immune	Epithelium, endothelium, stromal cells, α4β7 integrin-positive lymphocytes	IBD and lymphocyte homing	Barrier disruption plus MAdCAM-1 induction can be compared with each perturbation alone; immune recruitment is the output. Requires cytokine controls, endothelial rescue and immune-compartment benchmarking; immune recruitment should be interpreted as a candidate interaction, not a definitive dependency.	Sandborn et al., 2013; Miao et al., 2025; Guelfi et al., 2025
BBB	Brain endothelium, mural cells, neural-support compartment	BBB permeability and cerebral cavernous malformation	Node perturbations can be linked to permeability, transporter activity and spatial molecular-state outputs.	Bergmann et al., 2018; Dao et al., 2024
Brain tumour–neural	Glioblastoma cells, cerebral organoid neural tissue, candidate vascular/support nodes	Glioblastoma invasion and tumour–neural interaction	Tumour-cell perturbation, host-node perturbation and combined perturbation can test non-additive invasion or network outputs.	da Silva et al., 2018; Linkous et al., 2019; Krieger et al., 2020; Akhunbay-Fudge et al., 2026
Alveolar	AT2 cell, basement membrane, alveolar macrophage	PAP and lung infection	GM-CSF withdrawal tests macrophage maturation; pathogen exposure can test clearance output.	Kang et al., 2025
Glomerular	Podocyte, GBM surrogate, endothelium	Barrier dysfunction and proteinuria	Podocyte injury, matrix perturbation or complement challenge can be linked to filtration-barrier outputs.	Musah et al., 2017

AT2, alveolar type 2 cell; BBB, blood–brain barrier; ENS, enteric nervous system; GBM, glomerular basement membrane; GM-CSF, granulocyte-macrophage colony-stimulating factor; IBD, inflammatory bowel disease; ICC, interstitial cells of Cajal; LSEC, liver sinusoidal endothelial cell; MAdCAM-1, mucosal addressin cell adhesion molecule 1; MASLD, metabolic dysfunction-associated steatotic liver disease; PAP, pulmonary alveolar proteinosis; PHOX2B, paired mesoderm homeobox protein 2B; TTX, tetrodotoxin.

### Exemplar 1 – breakpoint mapping: intestinal–neuromuscular interface

iPSC-derived intestinal tissue incorporating enteric nervous system (ENS) progenitors develops a neuromuscular architecture that supports wave-like contractions, whereas intestinal organoids lacking ENS produce only isolated phasic contractions (Workman et al., 2017). Node-selective perturbation has identified at least three separable breakpoints within this chain: tetrodotoxin (TTX) abolishes propagating waves by blocking neural activity, Methylene Blue eliminates phasic rhythmicity consistent with interstitial cells of Cajal (ICC) function, and paired mesoderm homeobox protein 2B (PHOX2B) variants impair ENS differentiation itself (Workman et al., 2017). Complementary platforms – including three-germ-layer assembloids (Eicher et al., 2022) and co-differentiated organoids containing ENS, vasculature and smooth muscle (Childs et al., 2025) – now make it possible to compare breakpoint mapping across independently constructed systems. This example shows that chain dissection can localise single-node failures. However, it does not yet address whether concurrent failures interact, as they do in mesoscale maladaptation.

### Exemplar 2 – from coupling directionality toward maladaptation: hepatic sinusoidal interface

In the liver sinusoid, liver sinusoidal endothelial cells (LSECs) provide angiocrine signals, most notably WNT2, that promote hepatocyte differentiation, while hepatocyte-derived signals help maintain LSEC identity. Disruption of this reciprocal coupling leads to sinusoidal capillarisation, an early feature of chronic liver disease that contributes to stellate cell activation and fibrogenesis (DeLeve et al., 2004; Gracia-Sancho et al., 2021). iPSC-derived human liver bud organoids (HLBOs) provide an experimental platform for analysing this coupling. In an inverted multilayer air–liquid interface culture, hepatic endoderm, septum mesenchyme and sinusoidal progenitors self-organise to generate hepatocyte-like cells adjacent to sinusoidal endothelial cells (Saiki et al., 2025). Silencing WNT2 impairs both hepatocyte differentiation and sinusoidal endothelial network formation, indicating that this angiocrine signal supports function on both sides of the interface. This system provides an opportunity to move from coupling directionality to the third level of inquiry: does concurrent dysfunction of multiple nodes produce a non-additive disease phenotype? For example, sinusoidal capillarisation could be combined with hepatocyte lipotoxicity under metabolic stress, then compared with each perturbation alone. If fibrotic gene activation, hepatocyte dysfunction or endothelial-state change exceeds the pre-specified null expectation, the result would support experimental evidence for mesoscale maladaptation. Given its preserved sinusoidal interface and available perturbation tools, the HLBO platform is well suited to evaluating this hypothesis.

### Exemplar 3 – toward multi-node interaction: BBB and tumour–neural interfaces

Neural organoid and assembloid systems provide strong examples of interface-dependent coupling because barrier, vascular, neural and tumour compartments can be observed together. Forebrain assembloids established from dorsal and ventral spheroids recapitulate interneuron migration and functional integration, showing that patterned neural compartments can be fused and functionally coupled even when the interface is regional rather than multi-tissue (Birey et al., 2017; Xiang et al., 2017). Human BBB assembloids formed from brain and blood-vessel organoids acquire BBB-related molecular, transcriptomic and functional properties and perform neurovascular crosstalk (Dao et al., 2024). Importantly, patient-derived BBB assembloid models of cerebral cavernous malformation further show barrier breakdown and disease-relevant cellular changes (Dao et al., 2024). In chain-dissection terms, endothelial, mural-cell and neural-support compartments can be treated as candidate nodes within neural organoids or assembloids, while permeability, transporter activity and spatial molecular state provide respective interface outputs that can be tested singly and in combination.

Brain tumour organoid systems add a second neural example in which glioblastoma cells invade or interact with cerebral organoid tissue in real time (da Silva et al., 2018; Linkous et al., 2019; Krieger et al., 2020; Akhunbay-Fudge et al., 2026). A multi-node interaction test could compare vascular or neural-support perturbation alone, tumour-cell perturbation alone and the combined condition, asking whether invasion, barrier disruption or tumour–neural network formation exceeds the pre-specified null expectation. Together, BBB and tumour–neural assembloids extend the framework to interfaces at which barrier, vascular, neural and tumour compartments can be perturbed and observed in real time.

… understanding how simultaneously failing nodes interact requires an intact and functional interface and, therefore, depends on multi-tissue systems

## Opportunities and outlook

The detection of mesoscale maladaptation, defined as non-additive pathology arising from concurrent failure of multiple interface nodes, may represent one of the most distinctive contributions that multi-tissue organoid models can make to disease biology. Single-node breakpoint mapping remains valuable, but similar insights can sometimes be approximated using co-culture or conditioned-medium approaches. In contrast, understanding how simultaneously failing nodes interact requires an intact and functional interface and, therefore, depends on multi-tissue systems. Achieving this goal will require addressing several practical challenges. Node-selective perturbation is an ideal rather than a guarantee: pharmacological agents can have off-target effects, genetic perturbations may alter development, and inflammatory stimuli can affect several compartments at once. Chain dissection should, therefore, combine orthogonal perturbations, rescue experiments and lineage-resolved readouts wherever possible. Models lacking full systemic or immune compartments should be interpreted as controlled reductions rather than complete disease surrogates. Because many chronic diseases unfold over weeks to years, perturbation timing is part of the model rather than a technical detail. Acute perturbations are useful for identifying immediate coupling and causal direction, whereas chronic or repeated perturbations may better approximate inflammatory, metabolic or fibrotic remodelling. Sequential designs can test whether failure of node A sensitises node B, whereas simultaneous designs test whether concurrent failure creates a non-additive phenotype. Time-resolved molecular and functional readouts should, therefore, be selected to match the disease process being modelled. Finally, definitive multi-node interaction tests of mesoscale maladaptation remain uncommon. The immediate goal of this Perspective is, therefore, to make such tests explicit, reproducible and comparable across organoid platforms. It encourages a shift from asking “what did we build?” toward asking “what failed, where did it fail, and how do concurrent failures reshape the resulting phenotype?”
